# **G**ene-level **I**ntegrated **M**etric of negative **S**election (GIMS) Prioritizes Candidate Genes for Nephrotic Syndrome

**DOI:** 10.1371/journal.pone.0081062

**Published:** 2013-11-18

**Authors:** Matthew G. Sampson, Christopher E. Gillies, Wenjun Ju, Matthias Kretzler, Hyun Min Kang

**Affiliations:** 1 Pediatric Nephrology, University of Michigan, Ann Arbor, Michigan, United States of America; 2 Internal Medicine-Nephrology, University of Michigan, Ann Arbor, Michigan, United States of America; 3 Biostatistics, University of Michigan, Ann Arbor, Michigan, United States of America; Emory University School Of Medicine, United States of America

## Abstract

Nephrotic syndrome (NS) gene discovery efforts are now occurring in small kindreds and cohorts of sporadic cases. Power to identify causal variants in these groups beyond a statistical significance threshold is challenging due to small sample size and/or lack of family information. There is a need to develop novel methods to identify NS-associated variants. One way to determine putative functional relevance of a gene is to measure its strength of negative selection, as variants in genes under strong negative selection are more likely to be deleterious. We created a gene-level, integrated metric of negative selection (GIMS) score for 20,079 genes by combining multiple comparative genomics and population genetics measures. To understand the utility of GIMS for NS gene discovery, we examined this score in a diverse set of NS-relevant gene sets. These included genes known to cause monogenic forms of NS in humans as well as genes expressed in the cells of the glomerulus and, particularly, the podocyte. We found strong negative selection in the following NS-relevant gene sets: (1) autosomal-dominant Mendelian focal segmental glomerulosclerosis (FSGS) genes (p= 0.03 compared to reference), (2) glomerular expressed genes (p = 4×10^-23^), and (3) predicted podocyte genes (p = 3×10^-9^). Eight genes causing autosomal dominant forms of FSGS had a stronger combined score of negative selection and podocyte enrichment as compared to all other genes (p=1 x 10^-3^). As a whole, recessive FSGS genes were not enriched for negative selection. Thus, we also created a transcript-level, integrated metric of negative selection (TIMS) to quantify negative selection on an isoform level. These revealed transcripts of known autosomal recessive disease-causing genes that were nonetheless under strong selection. We suggest that a filtering strategy that includes measuring negative selection on a gene or isoform level could aid in identifying NS-related genes. Our GIMS and TIMS scores are available at http://glom.sph.umich.edu/GIMS/.

## Introduction

Minimal change disease [MCD] and focal segmental glomerulosclerosis [FSGS]) are rare forms of nephrotic syndrome (NS) with incidence rates of between 2-4/100,000/year in children in North America and the United Kingdom[1] and 1.4/100,000/year in adults from around the world[2].   The prevalence of known Mendelian forms of steroid resistant nephrotic syndrome (SRNS) (which is manifested most often as focal segmental glomerulosclerosis [FSGS], including congenital nephrotic syndrome, are estimated to be responsible for 1-80% of cases, and are highly dependent on age of onset and family history of NS[3]. With the increased application of sequencing technologies, rare single nucleotide variants (SNV) in genes that cause Mendelian forms of FSGS are being discovered at increasing rates[4-7]. In addition, common risk alleles for NS have also been identified in cohorts of sporadic NS using population-based methods[8,9] 

The current approach to gene discovery in NS focuses on using next-generation sequencing (NGS) in familial or sporadic NS, using targeted or whole exome sequencing[5,10]. From an analytic perspective, it is challenging to identify causal variants that emerge from these studies. In familial cases, variants are filtered by such means as mode of inheritance, population allele frequency, and predicted function of the variants. For sporadic cases, case-control association studies can also identify associated variants or genes. However, smaller sample size often limits the power to identify causal variants among a large pool of candidate variants beyond a statistical significance threshold. From a study design perspective, identifying additional parameters that can filter variants and/or improve power to detect statistically significant variants would be beneficial. Alternatively, due to the high cost of whole exome sequencing, effectively prioritizing lists of candidate genes or variants for sequencing could increase the power to identify novel functional variants associated with NS, given a limited budget. 

One way to prioritize putatively functional genes is to estimate the negative (or purifying) selection of a gene or a variant. Negative selection is the process by which deleterious variants that cause disease or reduce fitness are reduced or eliminated over generations [11]. It is widely known that common protein-altering (or non-synonymous) variants are depleted as compared to synonymous variants[12]. Because rare, protein-altering variants within functionally important genes are more likely to cause deleterious effects, they are under stronger negative selection than average genes. A number of established metrics are tightly correlated with negative selection. Comparative or functional genomics scores, such as PolyPhen-2 [13], GERP[14], and PhyloP [15] are correlated with negative selection *across* species. Negative selection *within* human populations can be estimated from allele frequency spectrum data obtained by population-scale sequencing[16]. 

We hypothesized that genes playing key roles in NS and glomerular diseases are under stronger negative selection compared to an average gene based on the following rationale. First, the low prevalence of NS, the high penetrance of NS-associated variants, and frequent early-onset cases of NS suggest that the genetic architecture of NS is more tightly coupled with negative selection than common complex diseases. Second, due to their specialized function and key role in homeostasis, glomerular- and podocyte-enriched genes are potentially under stronger negative selection compared to the average gene. If true, prioritizing candidate variants within genes under strong negative selection in familial or sporadic NS could enrich the proportion of truly causal variants. In addition, genes with glomerular or podocyte specific expression showing strong negative selection could be targeted, *a priori*, for a cost-effective custom sequencing study for large cohorts of affected NS subjects.

**Figure 1:**
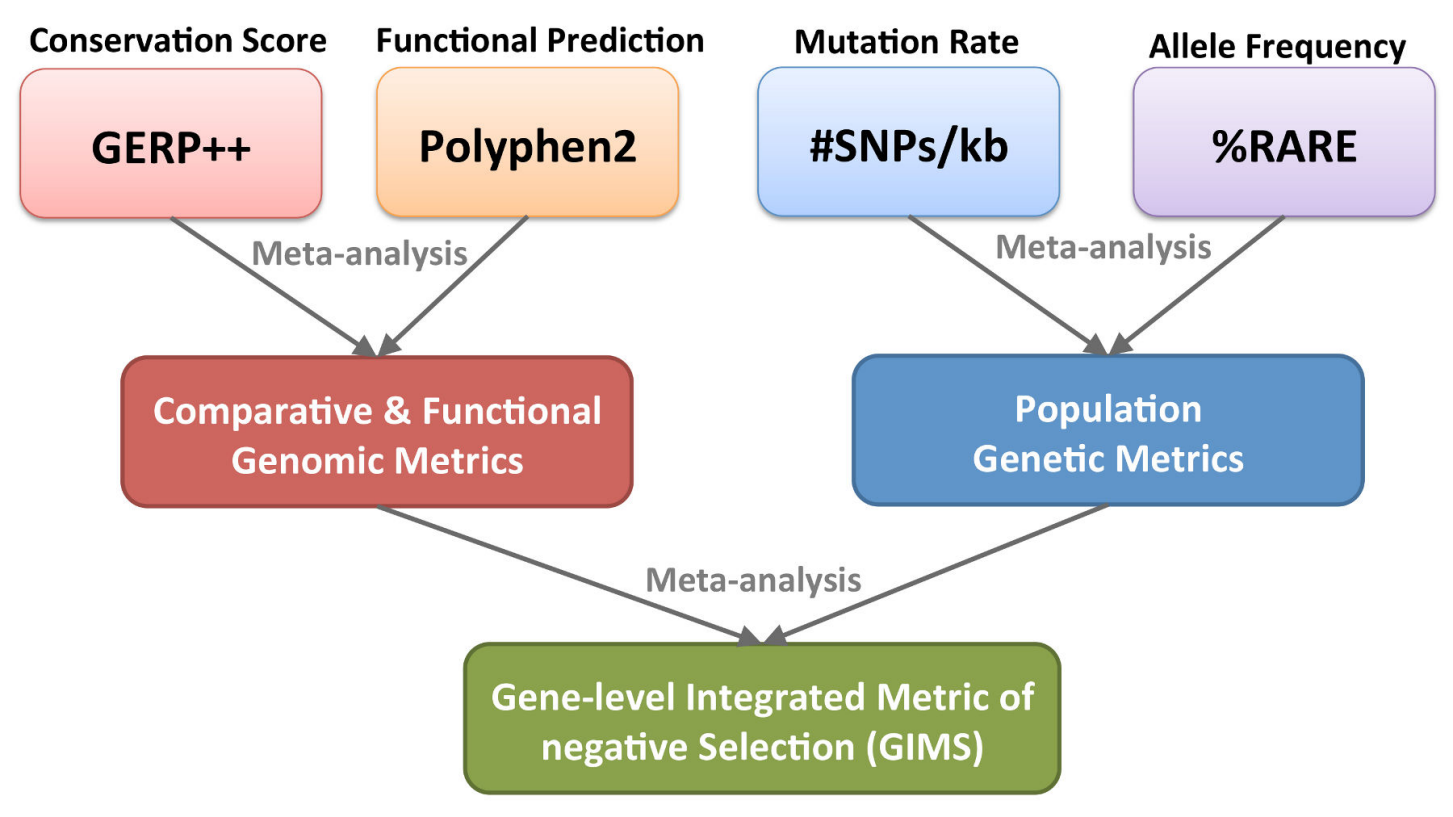
Overview of framework to generate GIMS score. Comparative genomic metrics (GERP++), functional genomic metrics (Polyphen2), and population genetic metrics (SNPs/kb and %RARE) from the 1000 Genomes Project were combined using meta-analysis into a single GIMS scores for 20,079 genes. Gene set enrichment analyses were then performed to evaluate the performance of GIMS scores and test for enrichment of selection in nephrotic syndrome relevant gene sets. 1000*G*=1000 Genomes Project; SNP/kb= Single Nucleotide polymorphisms/kilobase; FSGS=focal segmental glomerulosclerosis.

To test this hypothesis, we first used publically available comparative genomics and population genetics resources to create a genome-wide, **G**ene-level **I**ntegrated **M**etric of negative **S**election (GIMS) for each human gene (Figure **1**). Next, we applied this metric to known monogenic SRNS genes to characterize the negative selection properties of known NS-causing genes. We then applied GIMS to glomerular and podocyte expressed gene sets to test for enrichment of genes expressed in these NS-relevant cells. We characterized known autosomal dominant NS genes in terms of negative selection and podocyte specificity in order to further define a gene set that may be enriched for novel pathogenic NS variants. Finally, we created a **T**ranscript-level **I**ntegrated **M**etric of negative **S**election to characterize the strength of negative selection on an isoform level to provide an additional level of specificity beyond the GIMS score. 

## Materials and Methods

### Individual Metrics

Using the GenCODE database (v14) [21], we identified all autosomal genes that had coding sequence (CDS) ≥ 100 nucleotides. This resulted in the reference set of 20,079 genes. First, we examined all non-degenerate nucleotide positions in the CDS (ndCDS) and averaged comparative genomic GERP++[14] scores for each gene. Higher average GERP++ score implies stronger conservation on the gene. Second, we examined all possible single nucleotide variants (SNV) in the ndCDS and averaged the functional scores predicted by PolyPhen2 software [13] for each gene. The higher the score, the more likely a variant in the gene is causing deleterious amino acid changes. Third, we calculated the density of SNVs in the ndCDS for each gene from the whole genome sequencing of 1000 Genomes Phase 1 release[12]. Genes under stronger negative selection tend to show depleted mutation rate[17]. Finally, we calculated the fraction of common SNVs, defined as those with minor allele frequency (MAF) >0.5% for each gene, among the variants observed in the 1000 Genomes in the ndCDS. Under strong negative selection, we expect the fraction of common SNPs to be further depleted. We also avoided potential confounding due to gene length by ranking genes only based on average metric per gene, rather than using variance or p-values. The GERP++ scores and Polyphen2 scores were obtained from the dbNSFP database (version 2.0b4) [18]. 

### **G**ene-Level **I**ntegrated **M**etrics of negative **S**election (GIMS)

We integrated these comparative genomic, functional genomic, and population genetic metrics to estimate the enrichment of negative selection for each gene. Because the majority of genes are under negative selection, our goal was to assess whether a gene is under relatively stronger negative selection than the average gene rather than testing whether a gene is under negative selection compared to a neutral region. We ranked each gene based on each metric, and transformed the quantile of each gene into standard normal distributed z-score. We then combined the above four metrics stratified by CpG site into a single score hierarchically using Stouffer’s method. More specifically, we first combined conservation and functional scores and quantile-normalized them as ‘functional genomic metrics’. Similarly, we combined and quantile-normalized mutation rate and fraction of rare variants into ‘population genetic metrics’. These two metrics are again combined and quantile normalized to finally obtain a combined statistic for 20,079 genes (“GIMS Score”). GIMS expresses quantile across all genes, with a **lower** quantile being associated with **stronger** negative selection. The GIMS score for all genes is listed in Table **S1**. In our gene-level or gene-set analyses described below, we selected the longest transcript from GenCODE (v14) database for each gene, to avoid redundancy between transcripts sharing a large proportion of coding sequences. But we also produced Transcript-level Integrated Metric for Negative Selection (TIMS) score for 81,123 transcripts using the same method in Table **S2**.

### Gene Sets Utilized

**Figure 2:**
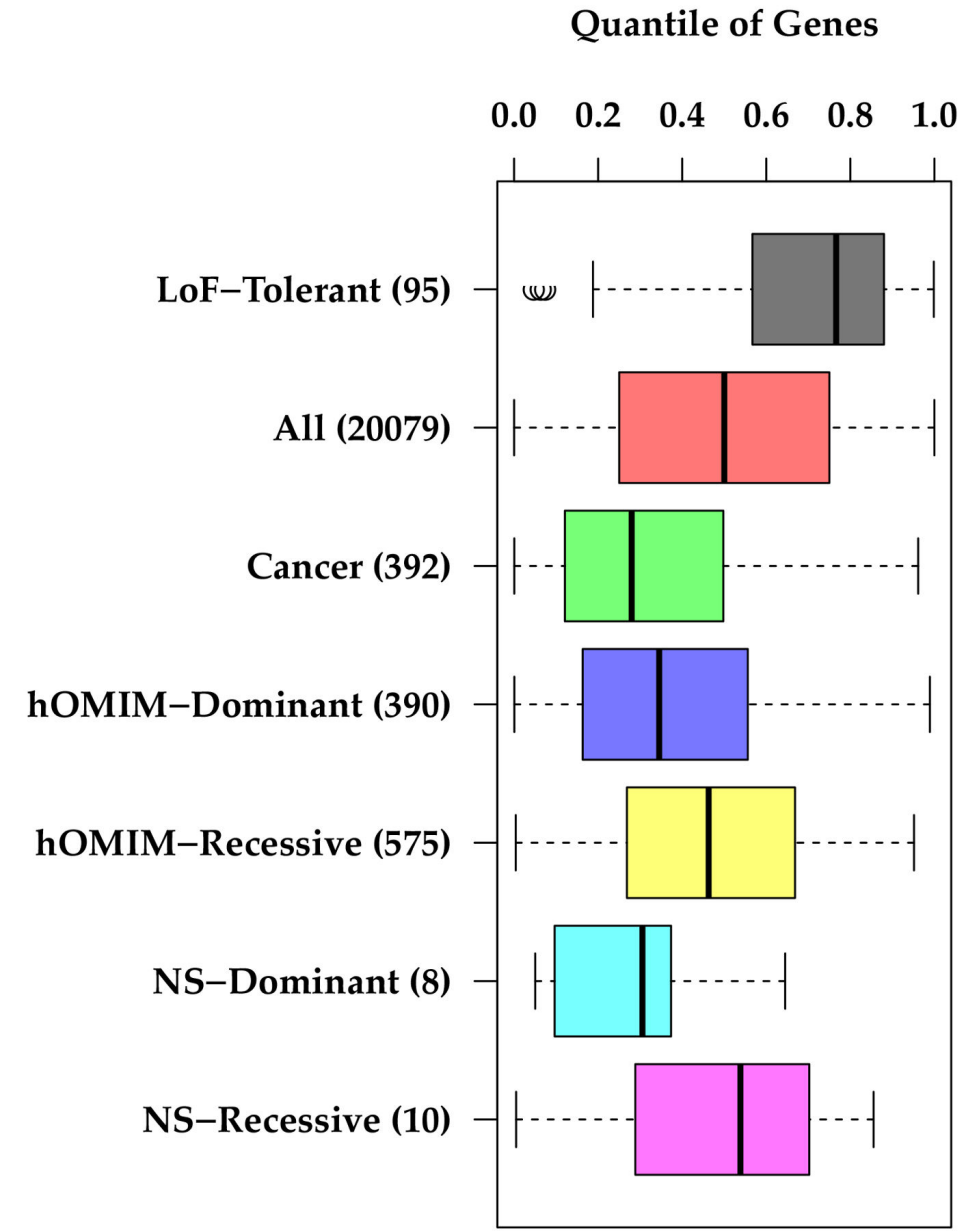
Distribution of GIMS score quantiles across gene sets with known functional categories “LoF-Tolerant” = genes containing common loss-of-function variants [19], “All”= 20,079 genes from GenCODE [20]. “Cancer”= genes registered in the catalogue of somatic mutations in cancer, “hOMIM-Dominant” and “hOMIM-Recessive” = dominant and recessive genes from hand-curated version of OMIM database [21], and “NS-Dominant” and “NS-Recessive” = genes in Mendelian forms of FSGS. *Note; lower GIMS score quantile=stronger negative selection.

To assess its accuracy, GIMS was initially applied to existing gene sets with known properties of selection; groups of genes with common loss-of-function variants seen in 1000G with no apparent deleterious affect (“LoF-Tolerant”) [19], genes implicated in cancer in humans (“Cancer”) [20], and genes associated with autosomal dominant and recessive Mendelian disorders from the hand-curated version of Online Mendelian Inheritance in Man database (“hOMIM-Dominant” and “hOMIM-Recessive”)[21] (Figure **2**). We expected higher GIMS scores for LoF-Tolerant genes (weaker negative selection), and lower scores for cancer and hOMIM genes (stronger negative selection) than a typical gene. 

**Figure 3:**
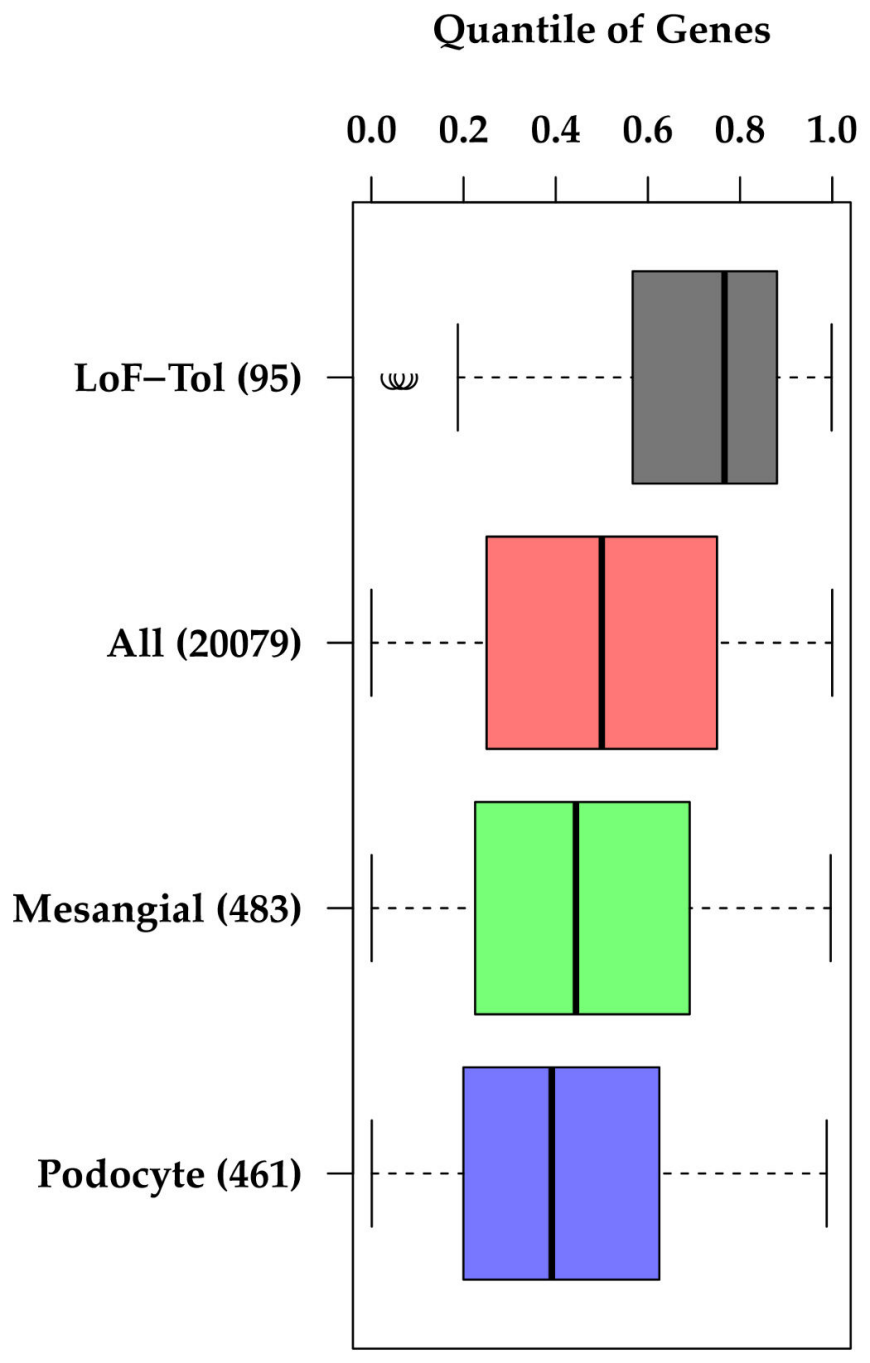
Distribution of GIMS score quantiles across gene sets specifically enriched in either glomerular or tubular compartment. “LoF-Tol” = Loss of Function tolerant gene set, “All”= 20,079 genes from GenCODE [20], “Glom”= genes with enriched expression in glomerular compartment, “Tubule”=genes with enriched expression in renal tubular compartment. *Note; lower GIMS score quantile=stronger negative selection.

**Figure 4:**
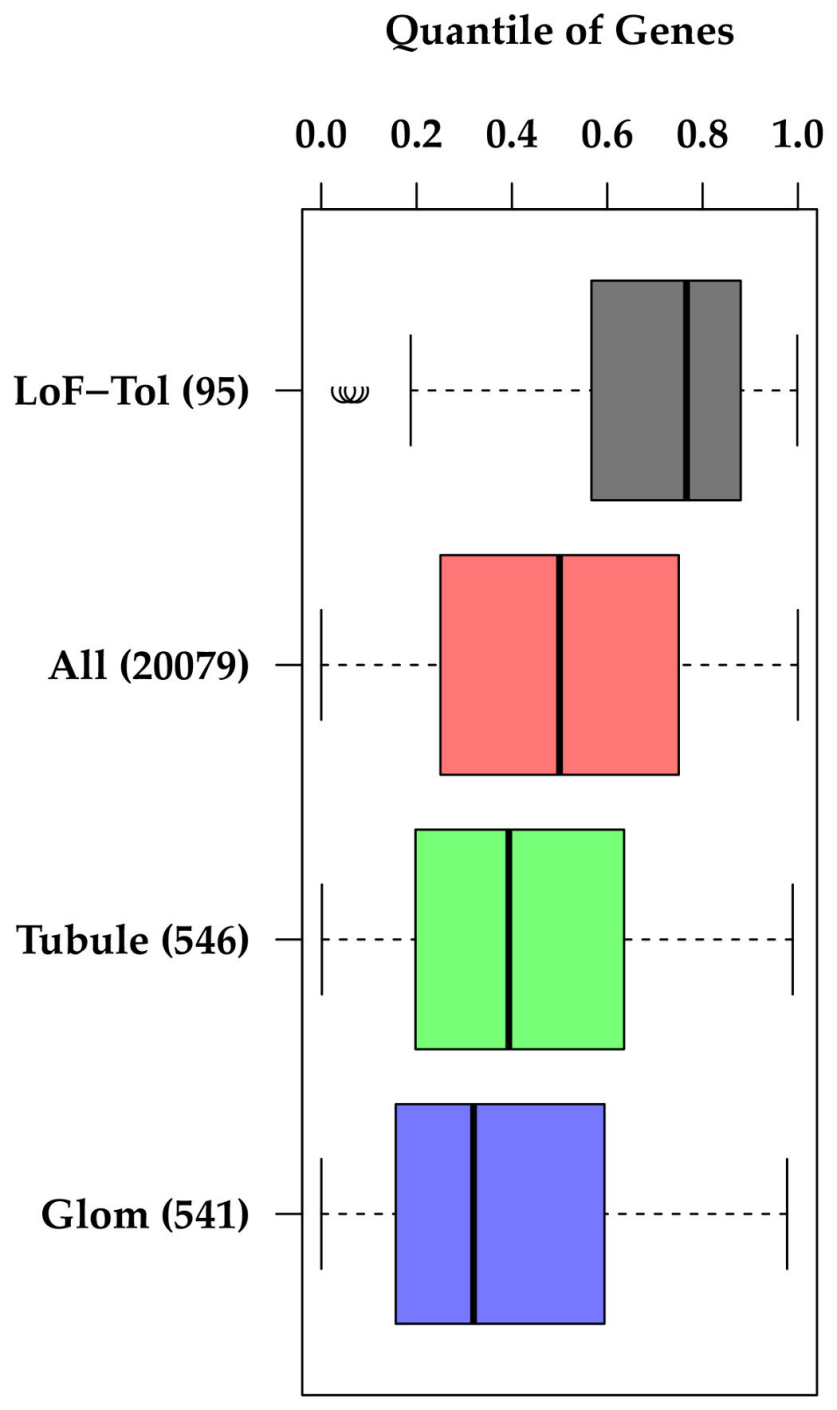
Distribution of GIMS score quantiles across gene sets predicted to be enriched in podocyte or mesangial cells. ** “LoF-Tolerant” and “All” genes are included as reference sets. *Note; lower GIMS score quantile=stronger negative selection.

Transitioning specifically to NS, 18 genes implicated in Mendelian forms of FSGS [22] were stratified by autosomal dominant and recessive mode of inheritance and specifically examined for their gene level properties of negative selection (Table **1**). To determine if genes within the glomerulus are enriched for negative selection, the top 2,000 most highly expressed genes in glomerular and tubular compartments of healthy kidney biopsy tissue from humans were identified from gene expression data from the European Renal cDNA Bank (microdissected into tubular and glomerular compartments)[23] (Figure **3**). Genes included in the top 2,000 list that were expressed only in one compartment were categorized as “glomerular only”, or “tubular only.” To determine cell lineage specific negative selection in the glomerulus, a podocyte-enriched gene set was compared to a mesangial-enriched gene set (Figure **4**)[24]. These cell-lineage specific gene sets were created by using a machine-based learning approach to analyze large numbers of kidney gene expression arrays after being trained with a group of podocyte gold standard positive and negative controls[24].

**Table 1:** List of the Mendelian FSGS genes studied for negative selection using GIMS.

Dominant Genes		Recessive Genes	
Gene Name	GIMS	Gene Name	GIMS
*WT*	.05	*MYO1E*	.005
*TRPC6*	.06	*PTPRO*	.04
*MYH9*	.14	*ITGB4*	.29
*ACTN4*	.29	*PLCE1*	.47
*LMX1B*	.32	*LAMB2*	.51
*ARHGAP24*	.33	*SCARB2*	.56
*CD2AP*	.41	*COQ6*	.67
*INF*	.64	*NPHS2*	.68
		*NPHS1*	.70
		*SMARCAL1*	.86

(Genes are stratified by mode of inheritance and GIMS score for each gene and geneset is presented). GIMS=Gene-level Integrated Metric of negative Selection.

### Gene set enrichment of negative selection

Distributions of GIMS score per gene set were illustrated using medians and interquartile ranges. P-values for the differences in distribution between gene sets were determined based on the Wilcoxon rank-sum test. The gene set of “All Genes” (n=20,079) was used as the reference, with a mean and median IMNS quantile of 0.5, by definition. Initially, we evaluated the differences in IMNS distribution between the reference and “LoF-Tolerant”, “Cancer”, “hOMIM-Dominant”, and “hOMIM-Recessive” gene sets, in addition to the sets of genes known to cause Mendelian forms of FSGS (“FSGS-Dominant”, “FSGS-Recessive”). This was followed by an evaluation of human kidney-biopsy derived gene sets, “glomerular-enriched expression (Glom)” and “tubular-enriched expression (Tubule)”. Finally, we evaluated the differences in GIMS score between human “podocyte-enriched (Podocyte)” and “mesangial-enriched (Mesangial)” gene sets. All gene sets were also compared to the reference gene sets. 

### Combined GIMS score and podocyte prediction score

**Figure 5:**
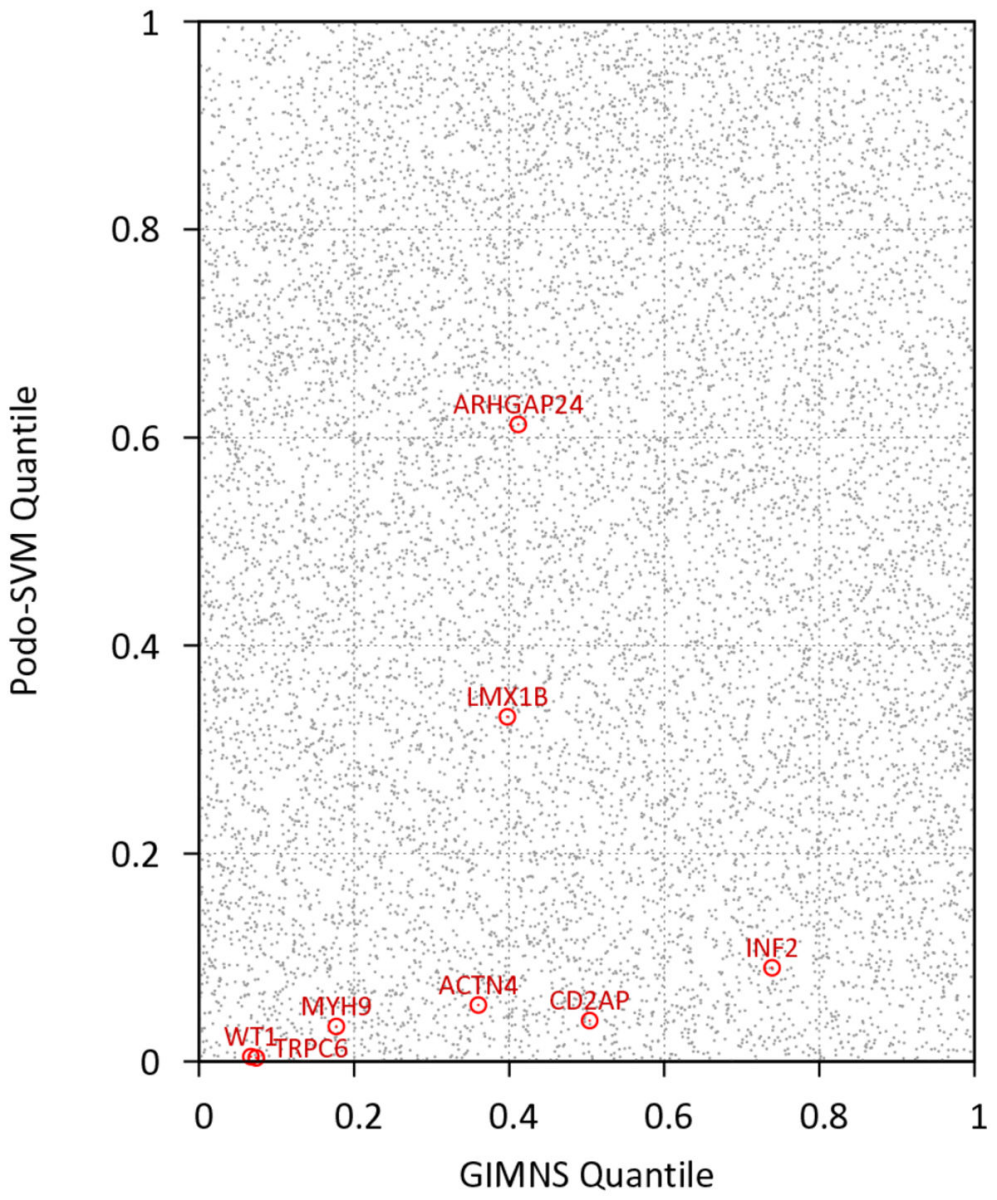
Scatter plot of GIMS score and Podocyte Prediction Score for 11,310 genes that had both metrics. Genes with stronger negative selection and higher predicted podocyte expression are located in the lower left quadrant of the plot. Known AD FSGS genes (red) are significantly more selected/enriched than all other genes. PODO-SVM=Podocyte prediction score.

We hypothesized that genes with the highest likelihood to contribute to NS would be those expressed in the podocyte and under strong negative selection, particularly those with dominant effects. We created a predicted podocyte enrichment score trained from Support Vector Machine (SVM) [24] after removing autosomal dominant NS genes from the training set, and then examined the distribution of GIMS and Podocyte Prediction scores in all genes that had both scores (Figure **5**). We specifically examined the six genes annotated in a recent review as causing autosomal dominant FSGS[22] as well as *LMX1B*[25] and *ARHGAP24*[26], which have also been associated with FSGS lesions inherited in an autosomal dominant manner. After combining the two scores using z-score based meta-analysis, we then used the Wilcoxon Rank-Sum test to examine whether those genes causing autosomal dominant FSGS had a stronger combined PODO/GIMS score as compared to all other genes. 

## Results

### Signature of negative selection in known gene sets

We first evaluated the distribution of the GIMS score on gene sets with known properties of negative selection (Figure **2**). As expected, loss-of-function tolerant genes [19] were under weaker negative selection than the average gene. The median quantile of GIMS score for these genes was 0.76, and its rank difference compared to the average gene (median=0.50) was significant with a p value of 7.8×10^-11^. The cancer gene set [20] was under strong negative selection with a median GIMS score of 0.28 and was significantly different from the average gene (p < 10^-30^). Among the gene sets reported to cause Mendelian disorders in the hand curated OMIM (hOMIM) database [27], we found significant difference in GIMS score between dominant and recessive disease genes. While the 390 dominant genes show strong enrichment of negative selection based on the distribution of GIMS scores (median quantile 0.35, p-value 3.3×10^-17^ compared to reference set), the 575 recessive genes showed only marginal enrichment (median quantile 0.46, p-value 9.7x10^-3^). The difference between dominant and recessive gene sets was also significant (p=1.2×10^-8^). Integrating multiple comparative genomic and population genetic resources overall increased the predictive power to identify genes under negative selection. For example, based on each individual metrics, the median quantile of cancer genes ranges from 0.30 to 0.39. The median quantile of the integrated GIMS score was 0.28. (Table **S3**) 

### Negative Selection in Mendelian FSGS Genes

We also evaluated 18 genes known to cause monogenic forms of FSGS, stratified by mode of inheritance. These genes, which cause significant disease that impacts fitness, are enriched for negative selection (Table **1**). The results were similar to the analysis of hOMIM dominant and recessive genes (Figure **2**). Eight FSGS genes that cause dominant forms of this disease have a median GIMS score of 0.30 (p= 0.03 compared to reference set), while ten genes that cause recessive forms have a median GIMS score of 0.54 (p= 0.88 compared to reference set). While this small set of recessive FSGS genes does not exhibit enrichment of negative selection as a whole, there is a wide range of negative selection in these ten genes, with two of ten recessive genes (*MYO1E, PTPRO*) under stronger negative selection than the most strongly selected dominant gene. 

In *INF2*, we observed that the metric of negative selection substantially differs in an isoform-specific manner. While the GIMS score of *INF2* based on the longest transcript was 0.64, a shorter isoform previously suggested to play an important role in FSGS (ENST00000398337.4) [28] showed a transcript-level score (TIMS) of 0.015, which is substantially stronger than those of the other isoforms (Table **2**). Similarly, shorter isoforms in *ARHGAP24* (ENST00000512201.1) and *LAMB2* (ENST00000494831.1) shows TIMS score of 0.014 and 0.063, which are much smaller than the gene-level scores, 0.33 and 0.51, respectively, which are based on the longest isoforms (Table **2**). 

**Table 2:** TIMS (Transcript-level Integrated Metric of negative Selection) score for all isoforms of *INF2, ARGHGAP24*, and *LAMB2*.

**Gene Name**	**Transcript ID**	**CDS Length (bp)**	**TIMS**
*INF2*	ENST00000481338.1	243	0.95
***INF2***	**ENST00000398337.4**	**702**	**0.015**
*INF2*	ENST00000330634.7	3720	0.72
*INF2*	ENST00000392634.4	3747	0.70
***ARHGAP24***	**ENST00000512201.1**	**246**	**0.014**
*ARHGAP24*	ENST00000509300.1	354	0.025
*ARHGAP24*	ENST00000503995.1	738	0.080
*ARHGAP24*	ENST00000514229.1	1866	0.44
*ARHGAP24*	ENST00000395183.2	1959	0.40
*ARHGAP24*	ENST00000264343.4	1965	0.48
***LAMB2***	**ENST00000494831.1**	**453**	**0.064**
*LAMB2*	ENST00000418109.1	5394	0.60

The transcript with strongest TIMS score are highlighted in bold

### Negative selection in glomerular-expressed genes versus tubular-expressed genes

We next evaluated whether genes with enriched expression in the glomerular compartment were under stronger negative selection than genes with enriched expression in tubulointerstitial compartment. To do this, we utilized gene expression information from glomerular and tubular compartments of healthy human biopsy tissue [23]. Of the top 2,000 most highly expressed genes in each of these two compartments, there were ~550 genes that were differentially expressed in a compartment-specific manner. Both of these genes sets were under significant negative selection as compared to the reference set of all genes, with median quantile of GIMS score of 0.32 and 0.39 for “Glomerular-Enriched” and “Tubular-Enriched”, respectively (Figure **3**). However, glomerular enriched genes were under significantly stronger negative selection than tubular enriched genes (p=5.0x10^-3^). The significance for enrichment of negative selection between glomerular enriched genes and the average gene had a p < 10^-22^.

### Negative selection in podocyte- versus mesangial-enriched genes

To determine whether negative selection was enriched in a cell-specific manner within the glomerulus, we evaluated gene sets composed of over 400 genes predicted to be enriched in either podocytes or mesangial cells [24]. While mesangial-enriched gene show a significant but weak enrichment of negative selection as compared to the reference gene set (median=0.44, p=3.0x10^-3^), podocyte genes show much stronger enrichment (median=0.39, p = 2.8x10^-9^) (Figure **4**). Comparison between podocyte and mesangial genes also show significant difference with a p=0.027.

### Combined GIMS-Podocyte prediction score

To determine if autosomal dominant FSGS genes shared similar characteristics of negative selection and podocyte specificity, we evaluated the combined rank of GIMS and podocyte prediction score for known AD FSGS genes (n=8) in the context of the ~11,310 genes that were scored using both metrics (Figure **5**). As a set, these eight genes had a significantly stronger combined score than all genes with a p-value of 1.3x10^-3^, median quantile of 0.10, and mean quantile 0.17 of combined score. As visualized in Figure 5, these autosomal dominant, monogenic NS genes clustered in a region with many other novel genes that share similar negative selection and podocyte specificity scores. 

## Discussion

We created a genome-wide metric of negative selection in order to determine if this metric, applied to nephrotic syndrome relevant gene sets, could serve useful in future gene discovery efforts for this disease. Our results demonstrate strong enrichment signatures of negative selection in the following gene sets: (1) genes causing dominant Mendelian form of FSGS, (2) glomerular-enriched genes, and (3) podocyte-enriched genes. We also discovered that AD FSGS genes had a stronger composite characteristic of negative selection and podocyte specificity than other genes. This suggests that genes with similar composite scores could represent higher priority candidates for investigation of their role in NS.

GIMS quantifies the strength of negative selection (a measure known to be associated with functional effects) by integrating diverse metrics across multiple mammalian species, multiple human populations, and multiple protein families. A strength of this score includes careful selection of the databases used to derive the GIMS score, which ensured that this single metric was computed from almost independent resources. Additionally, the metrics from each resource were carefully combined to avoid confounding effects, such as differential mutation rates at CpG dinucleotides or gene length. Finally, GIMS integrates multiple metrics of negative selection per gene into one score. This single score both improves efficiency and power and also facilitates easier comparison between genes. 

GIMS demonstrated consistent patterns of negative selection to the expectation for a group of gene sets that have established properties of strong enrichment or depletion of negative selection [19,21,27]. Encouragingly, the behavior of the “NS-Dominant” and “NS-Recessive” genesets mirrored those of “hOMIM-Recessive” and “hOMIM-Dominant” gene sets, thereby replicating the work of others who measured selection in Mendelian disorders using OMIM genes [21]. This supports the concept that those genes in which a single deleterious variant is sufficient to cause disease are under stronger evolutionary constraint than those genes that require two alleles to cause disease. Thus GIMS may be most useful in detecting variants that contribute to NS in a dominant manner. 

The creation and application of GIMS has provided a new insight on glomerular biology in terms of evolutionary selection. We tested for enrichment of negative selection in high quality gene sets composed of genes enriched for expression in the glomerular or renal tubular compartments, as well as mesangial or podocyte cells. We established that, as compared to tubular or mesangial gene sets, glomerular, and more specifically, podocyte gene sets, are under significant enrichment of negative selection. 

We posit a number of reasons that glomerular and podocyte genes are under stronger negative selection than tubular or mesangial cells. Podocytes are terminally differentiated cells with highly specialized substructures and functions. Deleterious variants that caused podocyte loss would be expected to have higher impact due to this cell’s inability to regenerate, and thus could more result in stronger selection against deleterious variants. This is also true in regards to variants that would affect overall glomerular function, as opposed to the regenerative abilities of the renal tubular epithelia. It may be that, as compared to tubular and mesangial genes, glomeruli and podocytes may lack the redundancy in genes or pathways that would compensate for the loss of function conferred by a deleterious variant.

As seen for *MYO1E and PTPRO*, there are genes associated with autosomal recessive forms of FSGS that are nonetheless under strong negative selection. One possibility for this is that heterozygous variants within these genes, distinct from those causing FSGS, may have an unrecognized contribution to other deleterious human diseases. We could speculate that perhaps these variants confer embryonic lethality and account for their strong negative selection, while those variants that cause FSGS are less deleterious in the context of negative selection.

It is possible that certain input data used to produce GIMS score, such as Polyphen 2 score, may have slightly biased our analysis in favor of stronger GIMS score in Mendelian genes, because Polyphen prediction uses known Mendelian variants as scores to train known pathogenic variants co-segregating with disease in Mendelian families, and those variants may contribute to stronger GIMS score for the gene. However, our GIMS score utilizes the average conservation and functional metrics across all protein coding sequences that are typically thousands of bases, so the potential confounding effect due to a limited number of pathogenic variants screened by Polyphen 2 score should be very small. Indeed, for dominant FSGS genes, we observed that conservation scores and population genetic metrics more strongly contributes to strong GIMS scores than Polyphen 2 scores. (Table **S3**)

Importantly, we observed that there are highly penetrant Mendelian nephrotic syndrome genes that do not display enrichment of negative selection, particularly those with autosomal recessive inheritance patterns. For instance, *NPHS1* and *NPHS2* have GIMS scores of 0.70 and 0.68, respectively. If we solely relied on GIMS, we would mask our ability to identify disease-associated variants within genes that are, overall, not enriched for negative selection. Thus we established a TIMS score and established that this transcript-level measure confers an ability to identify highly selected transcripts even if the GIMS score is not suggestive of overall negative selection on the genic level. Future studies to pursue should include studying negative selection on per-exon or per-nucleotide level. This would serve to increase sensitivity for detection of deleterious variants under negative selection, independently of gene- or transcript-level metrics. 

By combining GIMS score with a podocyte prediction score, we both visualized and quantified that known AD FSGS genes show enrichment of the orthogonal measures of strong negative selection and podocyte specificity. Thus, other genes in this stratum of selection and cell specificity could be viewed as high priority for future investigation in NS, via a variety of targeted strategies. 

In summary, we developed GIMS, calculated per gene, per gene-set scores, and per transcript scores, and have used this information to discover that podocyte and glomerular genes are under significant enrichment of negative selection. Our work has also defined GIMS and TIMS scores, genome-wide (Tables **S1** & **S2**). Given the critical, and pervasive, role of negative selection in human disease, we expect that this approach can be utilized for the discovery of contributory variants in many other diseases, particularly those with presumptive dominant inheritance. 

An interactive version of Figure 5 is available as a web application at http://glom.sph.umich.edu/GIMS/ and allows the user to either search by gene name for GIMS and TIMS scores or to search the figure in a region of interest. In addition, the GIMS and TIMS scores are available as downloadable files from the same website. 

## Supporting Information

Table S1 **GIMS scores derived for 20,079 human genes.**
(XLS)

Table S2 **TIMS scores derived for 81,123 transcripts.**
(XLS)

Table S3 **Pairwise p-value of Wilcoxon’s rank-sum test between different groups of genes based on GIMS scores and each individual metric used for constructing GIMS score.**
(XLS)
